# First Record of Triplet Embryos in Sea Turtles: Evidence of Natural Polyembryony in a Wild Green Turtle (*Chelonia mydas*) Nest From Guanahacabibes, Cuba

**DOI:** 10.1002/ece3.74382

**Published:** 2026-09-28

**Authors:** Julia Azanza‐Ricardo, Annelisse Bárcenas‐Ibarra, Kevin Armando Domínguez‐León, Randy Calderón‐Peña, Yunaisy Guerrero‐Esquijarosa, Eduardo E. Colin‐Hidalgo, José A. Marmolejo‐Valencia, Horacio Merchant‐Larios, Daniel J. Sánchez‐Ochoa, Horacio de la Cueva

**Affiliations:** ^1^ Centro de Investigaciones Marinas Universidad de La Habana Havana Cuba; ^2^ Biodiversidad, Conservación e Investigación A.C. Nuevo León Mexico; ^3^ Facultad de Biología Universidad de La Habana Havana Cuba; ^4^ Parque Nacional Guanahacabibes, Centro de Investigación y Servicios Ambientales (ECOVIDA) Pinar del Río Cuba; ^5^ Departamento de Biología Celular y Fisiología Instituto de Investigaciones Biomédicas, UNAM Mexico City Mexico; ^6^ Facultad de Estudios Superiores Iztacala‐UNAM Estado de Mexico Mexico; ^7^ División de Biología Experimental y Aplicada CICESE Ensenada Baja California Mexico

**Keywords:** *Chelonia mydas*, congenital malformations, embryonic development, polyembryony, sea turtles, triplet embryos

## Abstract

Polyembryony is an exceptionally rare developmental phenomenon in reptiles and is typically documented as isolated cases of monozygotic twinning. Evidence of triplet embryos in chelonians is particularly scarce and has previously been reported only in managed or captive conditions. We report and describe an exceptional case of polyembryonic triplet embryos from a wild green turtle ( *Chelonia mydas* ) nest on the Guanahacabibes Peninsula, Cuba—representing one of only two confirmed chelonian triplet records and the first documented under natural incubation conditions in a sea turtle nest. The nest achieved 91.6% hatching success and normal hatchling performance; three of the 11 unhatched eggs (27.3%) contained embryos with congenital malformations, including a polyembryonic triplet embryo configuration. Morphological assessment, developmental staging, and yolk sac examination demonstrated three embryos attached to a single continuous yolk mass, each possessing an independent vitelline vascular network, consistent with a monozygotic origin. Marked developmental asymmetry and craniofacial malformations in one embryo are compatible with resource limitation and asymmetric sibling competition within a shared yolk environment. Histological analysis of urogenital tissues revealed ovarian‐type gonads in all three embryos, consistent with the expected female‐biased sex ratios at higher incubation temperatures. These findings provide robust evidence of polyembryony under natural incubation conditions and highlight the developmental constraints inherent to multi‐embryo development in sea turtles, underscoring the importance of long‐term nest monitoring for detecting rare embryological events.

## Graphical abstract

A rare case of polyembryonic triplets in a wild green turtle ( *Chelonia mydas* ) nest demonstrates how multiple embryos can develop within a single egg under natural conditions. Morphological and embryological evidence, including independent yolk vasculature and marked asymmetry among embryos, support a monozygotic origin and highlight developmental constraints in shared‐resource systems. These findings provide new insight into developmental instability and the limits of embryonic plasticity in natural reptile populations.

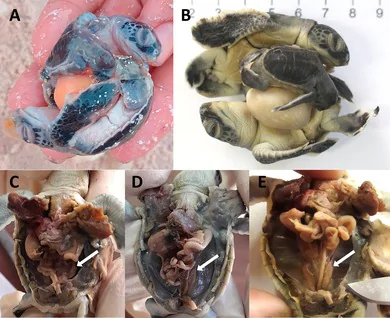

## 1 Introduction

Polyembryony—the development of two or more embryos from a single fertilized egg—produces identical siblings, such as monozygotic twins, triplets, or higher‐order multiples (Patterson 1927; Avise 2012). Marchal (1904) recognized three forms of polyembryony: (1) experimental polyembryony, involving artificial induction of multiple embryos; (2) sporadic (teratological) polyembryony, describing the occasional occurrence of multiple embryos in species that are normally monembryonic; and (3) specific (obligate or constitutive) polyembryony, in which multiple embryos are produced consistently as part of the species' reproductive biology.

Sporadic polyembryony occurs across invertebrate and vertebrate taxa and most commonly manifests as monozygotic twins, although higher‐order multiples have also been reported (Patterson 1927; Avise 2015). It has been recorded across diverse invertebrate groups—including cestodes, cnidarians, echinoderms, annelids, and arthropods—yet most well‐documented cases in the literature involve vertebrates (Patterson 1927; Craig et al. 1997; Iwabuchi 2019). Despite its broad phylogenetic distribution, sporadic polyembryony remains rare. The best‐known example is monozygotic twinning in humans, for which the global twinning rate is 12.0 twin deliveries per 1000 deliveries (Monden et al. 2021). Across other vertebrates, such events are frequently associated with high embryonic or neonatal mortality (Iwabuchi 2019), and the developmental mechanisms and evolutionary implications of polyembryony in oviparous vertebrates remain poorly resolved.

In reptiles, documented cases of polyembryony are uncommon and primarily involve monozygotic twins, with triplets reported only rarely in lizards, snakes, and crocodilians (Shine 1985; Krauss and Horn 2004; Eckhardt 2018; Nečas et al. 2020; Vega and Siroski 2021; Santiago et al. 2025). Within Cuba, a naturally occurring case of reptilian polyembryony has also been documented in the Cuban boa ( *Chilabothrus angulifer* ), involving monozygotic twins (Rodríguez‐Cabrera et al. 2022). These observations indicate that polyembryony in reptiles is best regarded as a rare developmental anomaly detected opportunistically rather than a routinely studied reproductive phenomenon; usually identified incidentally during nest excavations or post‐hatching examinations rather than through targeted investigation.

Evidence of polyembryony in chelonians is scarce. Most reported cases involve twin embryos, documented mostly in captive freshwater turtles and tortoises, with only isolated records from wild sea turtle populations, including the olive ridley turtle ( *Lepidochelys olivacea* ), the leatherback turtle ( *Dermochelys coriacea* ), the loggerhead turtle ( *Caretta caretta* ), the hawksbill turtle ( *Eretmochelys imbricata* ), and the green turtle ( *Chelonia mydas* ) (Eckert 1990; Tucker and Janzen 1997; Azanza et al. 2008; Piovano et al. 2011; Bárcenas‐Ibarra et al. 2015; Feitosa et al. 2025). Documented triplet embryos in chelonians are exceptionally rare. The only other confirmed report we could find describes triplet hatchlings of the freshwater turtle ( *Podocnemis unifilis* ) under managed conditions (Cunha et al. 2024). However, that case lacked embryonic staging, yolk sac vascular characterization, and detailed morphological analysis, limiting inferences regarding the embryos' developmental origins.

We report polyembryonic triplet embryos from a wild green turtle nest on the Guanahacabibes Peninsula, Cuba. To our knowledge, this represents one of only two documented cases of triplets in chelonians, and the first recorded in a wild sea turtle nest. By integrating developmental staging, yolk sac vascular analysis, detailed morphological assessment, and histological sex identification, this study provides the most comprehensive embryological documentation of polyembryony in a sea turtle to date.

## 2 Methods

The green turtle nest was monitored as part of a long‐term sea turtle monitoring program conducted by staff from the University of Havana, trained volunteers, and the administration of Guanahacabibes National Park. Natural hatchling emergence was observed during a routine nighttime patrol. Fifteen hatchlings were randomly selected during emergence for external examination, morphometric measurements, and performance assessment. Each hatchling was carefully examined for congenital malformations. Straight carapace length (SCL) and straight carapace width (SCW) were measured using a Frankford Arsenal 150‐mm digital caliper (±1 mm), and curved carapace length (CCL) and curved carapace width (CCW) were measured using a flexible measuring tape; means and standard deviations (±SD) are reported. All 15 hatchlings were subsequently released on the beach to reach the sea naturally.

Nest vitality was assessed by evaluating the performance of these 15 hatchlings using self‐righting and locomotor tests following the methodology described by Booth et al. (2013). Self‐righting performance was assessed as the time required for an inverted hatchling to return to an upright position, whereas locomotor performance was measured as the distance traveled over a one‐minute period; means and standard deviations (±SD) are reported.

The nest was excavated on 23 August 2025, after the observed natural emergence and completion of the incubation period (≈60 days; Calderón‐Peña et al. 2020). Following standard protocols (Miller 1999), the nest inventory included counts of eggshells, live and dead hatchlings, eggs with obvious embryonic development, and eggs without obvious embryonic development to determine clutch size, hatching success, and emergence success. All hatchlings remaining in the nest (alive or dead) at the time of excavation were examined for congenital malformations, as malformed hatchlings, even those with minor malformations, may fail to emerge from the nest (Bárcenas‐Ibarra et al. 2015). Any live hatchlings found during the nest inventory were subsequently released on the beach to reach the sea naturally. Nest‐site characteristics, including distance from the high‐tide line and shading level, were also recorded. Incubation temperature was not measured directly within the study nest. As a local thermal reference, temperature data were obtained from a Pendant 8K‐UA‐001‐08 data logger placed at a depth of 50 cm within a nearby nest located 8 m from the study nest. Temperature was recorded at 2‐h intervals throughout the incubation period.

All unhatched eggs were opened, and those containing embryos were examined for congenital malformations; an embryo was defined as any organism present within an unhatched egg at any developmental stage. For descriptive purposes, the three unhatched eggs containing embryos were labeled as Egg A, Egg B, and Egg C, based on the number of embryos identified within each egg (single, twin, and triplet embryos, respectively). All malformed organisms were photographed under ambient lighting conditions. When multiple congenital malformations occurred in a single organism, each malformation was recorded separately for subsequent taxonomic classification (Bárcenas‐Ibarra et al. 2015). Embryonic developmental stage was determined following Miller (1985).

Detailed morphological examinations were conducted on all malformed embryos. The same carapace measurements described above for hatchlings (SCL, SCW, CCL, and CCW) were obtained using the same procedures and instruments. In addition, embryo mass was measured using a Rhino digital balance (±0.2 g).

Polyembryony was evaluated through macroscopic examination of the yolk sac and associated vasculature during embryo necropsy. Vascular emergence points, branching patterns, and spatial orientation of vitelline vessels relative to each embryonic axis were examined to assess the independence of embryonic circulatory systems (see Phillott and Godfrey 2020).

Urogenital tissues were dissected from egg C, which contained three embryos. Histological examination was limited to these three embryos due to the availability of preservation materials at the time of collection; the remote location of the study site precluded obtaining additional supplies within the required timeframe. The tissues were preserved in 70% ethanol. Although ethanol is not an optimal fixative for histological preservation of embryonic gonads, the dorsal exposure of the urogenital tissues allowed successful dissection and subsequent processing. Dissected tissues were then fixed in paraformaldehyde 4% for 24 h and washed in 1× PBS for 12 h. For paraffin embedding, tissues were dehydrated through a graded ethanol series (75%–100%) followed by xylene. Serial sections (10–13 μm thick) were obtained with a microtome (820, American Optical, USA) and stained with Periodic Acid–Schiff, then counterstained with hematoxylin. Gonadal sex was determined according to established histological criteria for sea turtle embryos (Merchant‐Larios 1999).

All examinations and necropsies were conducted under controlled field conditions using sterile instruments. All procedures complied with institutional guidelines and were performed under authorization from the relevant environmental authorities as part of an established conservation and monitoring program.

## 3 Results

The evaluated nest represented 1.67% of the total number of nests analyzed during the season (*n* = 60) and 0.07% of all nests historically exhumed in the study area (*n* = 1360). The nest was located 3.5 m from the high‐tide line and fully exposed, with no shading. The mean incubation temperature recorded in a nearby nest located 8 m from the study nest was 31.87°C ± 1.03°C. Of the 131 eggs deposited, 120 developed and hatched (91.6%), eight eggs showed no evidence of embryonic development (6.1%), and three unhatched eggs contained embryos that died before hatching (2.3%). No live or dead hatchlings remained in the nest at the time of excavation. Accordingly, both hatching and emergence success were 91.6%.

The 15 hatchlings evaluated during natural emergence exhibited normal vitality. Mean self‐righting time was 2.1 ± 0.8 s, and mean locomotor performance was 6.9 ± 1.4 m/min.

Necropsy of the three unhatched eggs revealed embryos at different developmental stages, all presenting congenital malformations. One egg contained a single embryo (Egg A), one contained twin embryos (Egg B), and one contained triplet embryos (Egg C).

Egg A contained a single embryo at developmental stage 28 (Miller 1985) and exhibited supernumerary vertebral scutes on the carapace, with seven scutes rather than the typical configuration of five in *C. mydas* (Pritchard and Mortimer 1999; Figure 1A).

**FIGURE 1:**
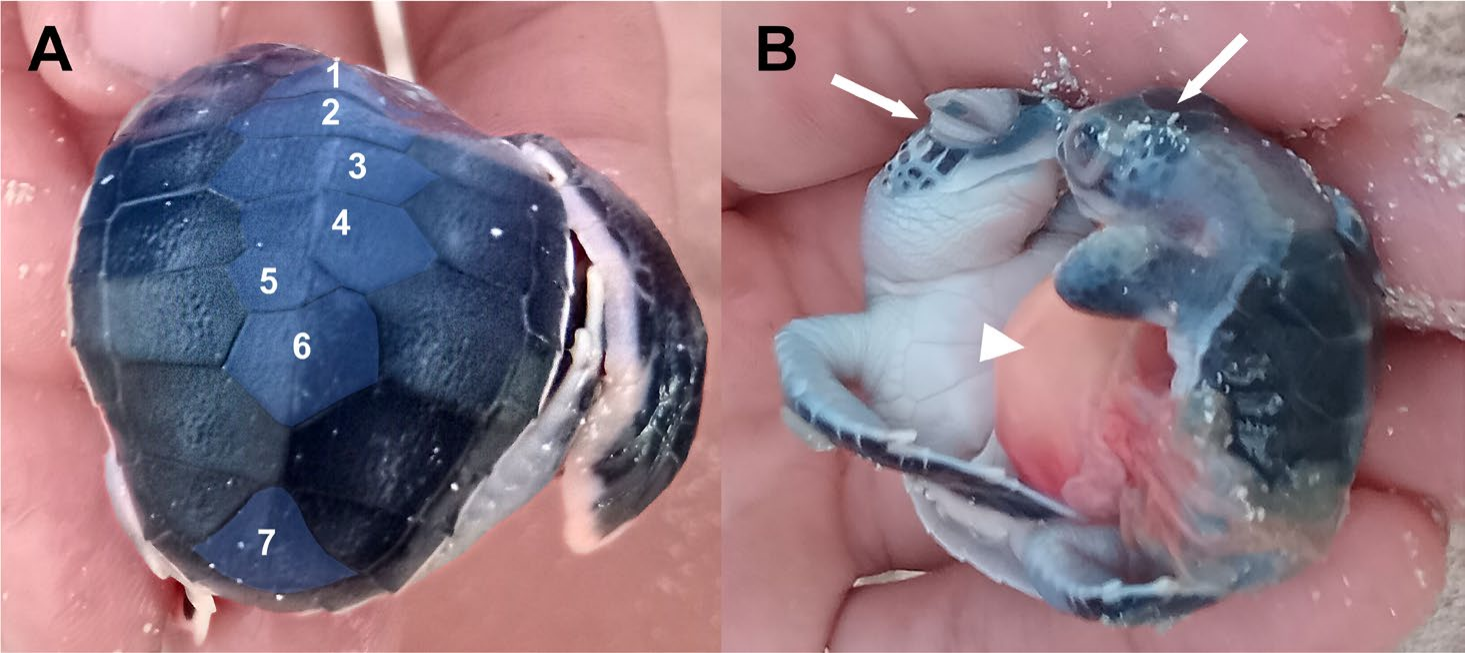
External morphology of embryos from Eggs A and B. (A) Single embryo from Egg A exhibiting supernumerary vertebral scutes on the carapace (dorsal view). (B) Polyembryonic twin embryos from Egg B attached to a single continuous yolk mass (arrowhead). Arrows indicate head position.

Egg B contained two embryos attached to a single continuous yolk mass (Figure 1B). Embryo B1 was classified as stage 28 (Miller 1985) and was larger than embryo B2 (stage 27; Miller 1985). Both embryos exhibited supernumerary vertebral scutes, with six scutes each rather than the typical five. No somatic fusion or shared body structures were observed; both embryos had independent body axes and organogenesis.

Egg C, presented in Figure 2, contained three embryos attached to a single continuous yolk mass. No somatic fusion or shared structures were observed. Each embryo displayed a distinct body axis and organogenesis. Macroscopic examination of the yolk sac during necropsy revealed three independent vitelline vascular networks, each originating from a different embryonic axis and exhibiting distinct branching patterns across the yolk surface.

**FIGURE 2:**
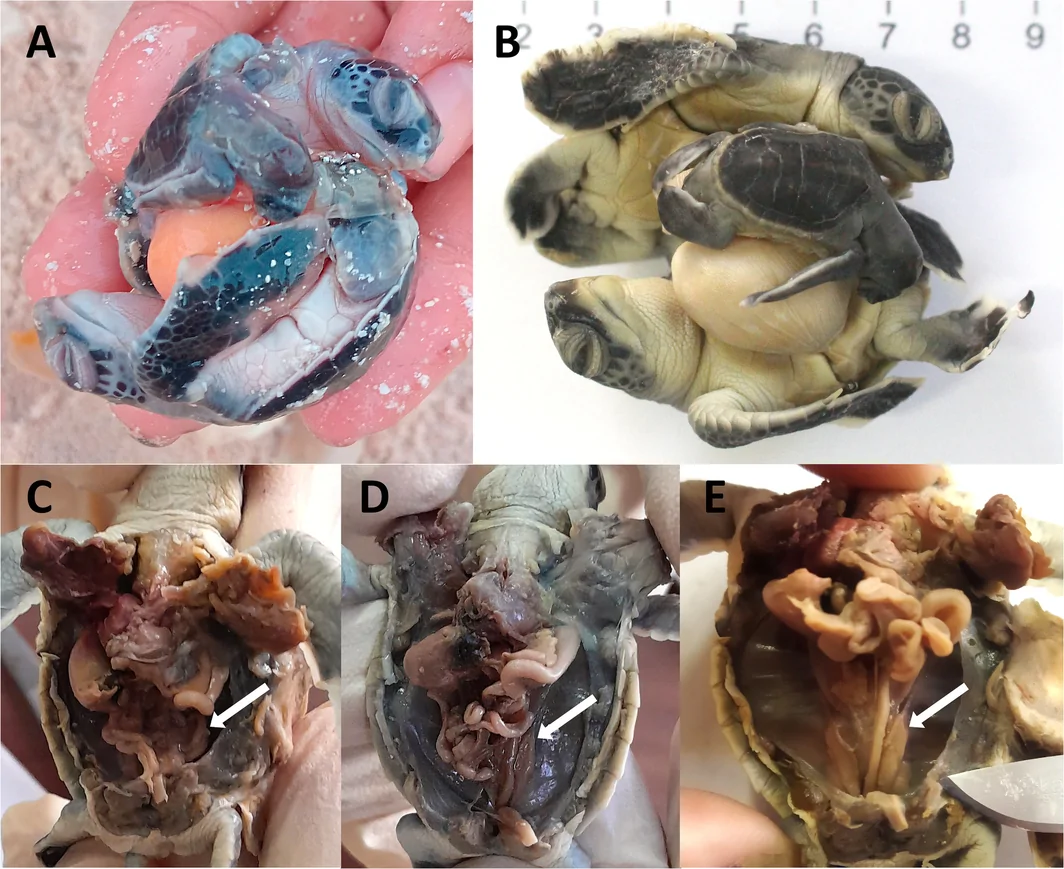
Structural configuration of polyembryonic triplet embryos from Egg C. (A) The condition of three embryos attached to a single continuous yolk mass immediately after extraction from Egg C. (B) Laboratory view of embryos C1, C2, and C3 prior to necropsy, illustrating their spatial arrangement around a common yolk mass. (C–E) Individual necropsy views of embryos C1, C2, and C3, respectively, showing distinct body axes and differentiated internal organ structures (arrows indicate the gonad–kidney complex).

Embryos C1 and C2 were classified as stage 28 (Miller 1985) and were similar in size and mass (Figure 3; Table 1). Both exhibited supernumerary vertebral scutes, with six each rather than the typical five. Embryo C3 was smaller (stage 27; Miller 1985) and presented supernumerary vertebral scutes, with six rather than the typical five, as well as additional craniofacial malformations, including microcephaly, left anophthalmia, right macrophthalmia, and mandibular hypoplasia (Table 1; Figure 4).

**FIGURE 3:**
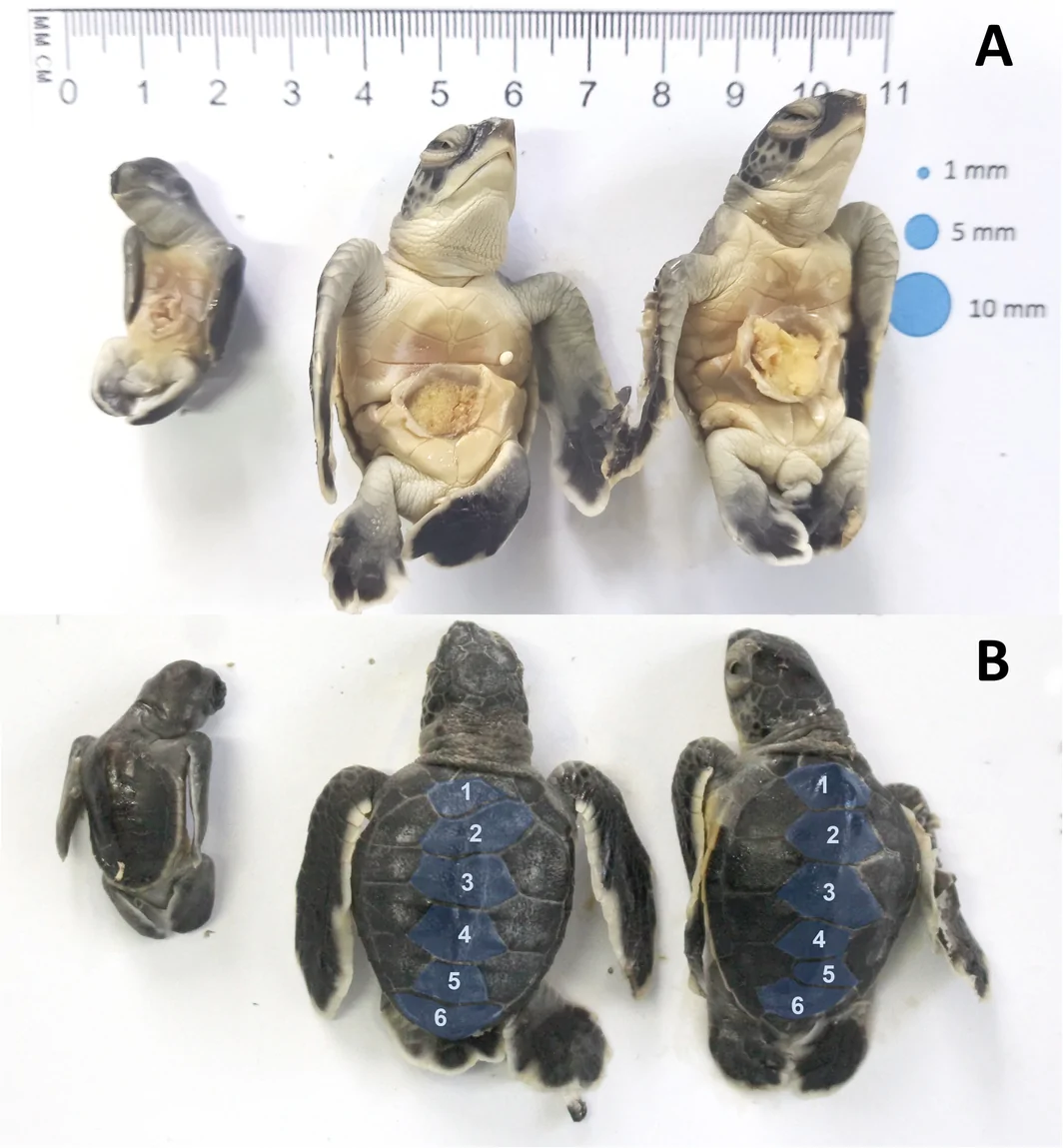
Comparative dorsal and ventral morphology of polyembryonic triplets from Egg C. (A) Dorsal view of embryos C3, C2, and C1 (left to right), illustrating relative size disparity among embryos and the presence of supernumerary vertebral scutes in embryos C1 and C2. (B) Ventral view of the same embryos showing overall external morphology and proportional differences among individuals.

**TABLE 1:** Morphometric measurements and body mass of polyembryonic triplet embryos (Egg C) and hatchlings from a naturally incubated nest of *Chelonia mydas* (131 eggs) in Guanahacabibes Peninsula, Cuba.

Individual	Sex	Mass (g)	CCL (mm)	SCL (mm)	CCW (mm)	SCW (mm)
Embryo C1	Female	14.2	40	33	31	25
Embryo C2	Female	15.1	40	32	32	24
Embryo C3	Female	5.6	23	18	15	12
Total embryo mass (Egg C)	—	34.9	—	—	—	—
Shared yolk mass	—	8.1	—	—	—	—
Hatchlings (mean ± SD, *n* = 15)	Unknown	—	57.1 ± 2.1	49.6 ± 2.1	47.6 ± 1.1	37 ± 1.2

*Note:* Embryos from the triplet egg were attached to a single continuous yolk and therefore shared the same yolk resource.

Abbreviations: CCL, curved carapace length; CCW, curved carapace width; SCL, straight carapace length; SCW, straight carapace width.

**FIGURE 4:**
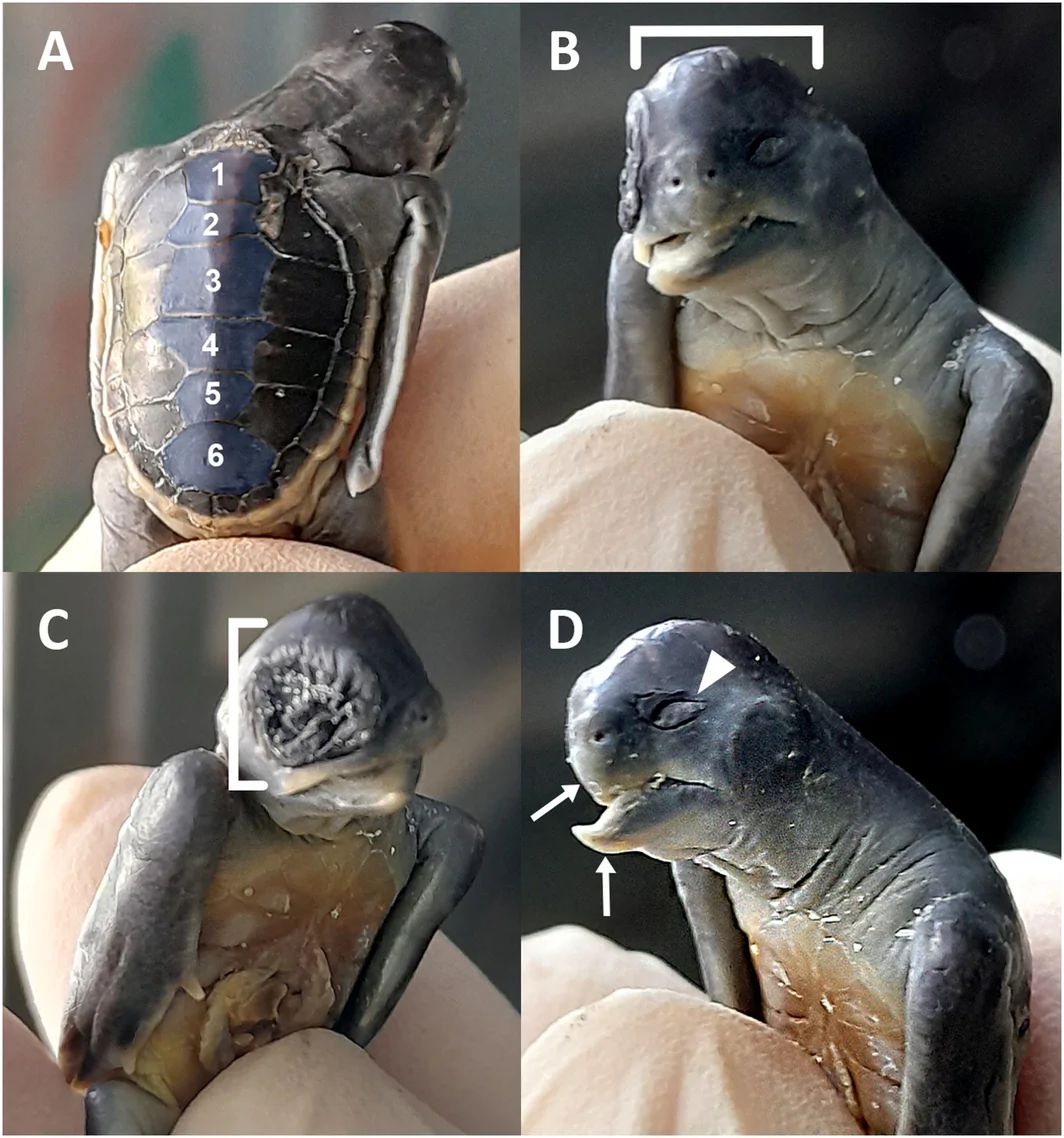
External congenital malformations in embryo C3 from Egg C. (A) Dorsal view showing supernumerary vertebral scutes. (B) Frontal view showing microcephaly (bracket). (C) Right lateral view showing right macrophthalmia (bracket). (D) Left lateral view showing left anophthalmia (arrowhead) and mandibular hypoplasia (arrows). Orientation is shown with the plastron facing downward and the carapace upward.

Histological examination identified all three Egg C embryos as female, using the presence of a conspicuously thickened columnar surface epithelium (i.e., the ovarian cortex), which is separated from the medullary zone by a basal membrane (dashed blue line in Figure 5).

**FIGURE 5:**
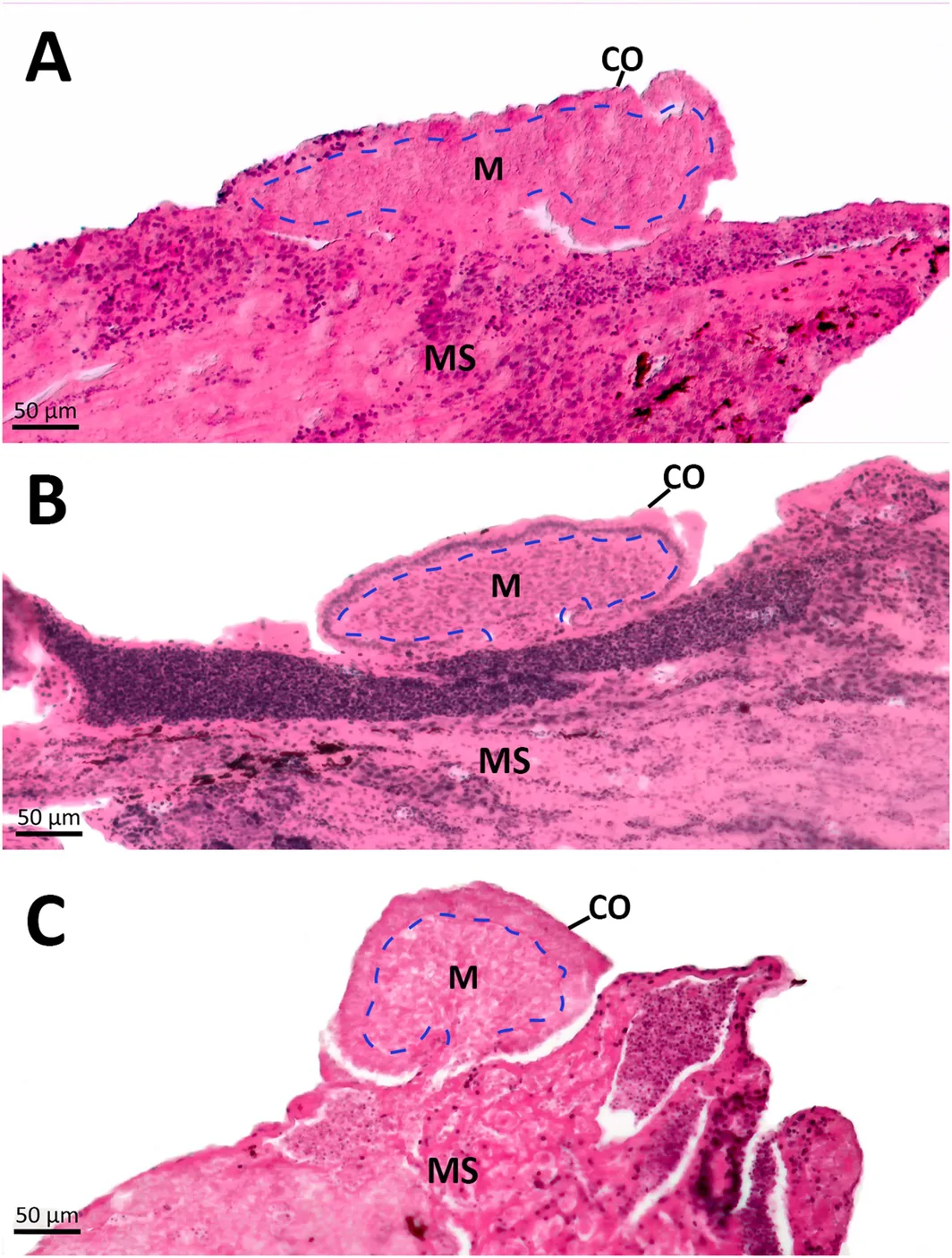
Histological sections of urogenital tissue from Embryo C1 (A), Embryo C2 (B), and Embryo C3 (C), obtained from Egg C. All sections exhibit ovarian differentiation characterized by a conspicuous cortical zone (CO) clearly separated from the medullary region (M). The dashed blue line indicates the boundary between cortex and medulla. MS = mesonephric tissue.

## 4 Discussion

The occurrence of polyembryonic twins and triplet embryos within a single naturally incubated *Chelonia mydas* nest represents an exceptionally rare developmental event. To our knowledge, the triplet embryos described here constitute the first record from a wild sea turtle nest and only the second documented case in any chelonian. This observation extends the known spectrum of naturally occurring polyembryony in reptiles and provides a unique opportunity to examine the developmental processes underlying this phenomenon.

Monozygotic twinning has been reported in several sea turtle species; most records correspond to isolated observations and do not provide reliable estimates of frequency. Studies based on large sample sizes indicate that twinning is consistently rare across sea turtles, generally occurring at frequencies below 0.1% (Bárcenas‐Ibarra et al. 2015). In green turtles, reported prevalence ranges from 0.01% to 1.45%, although the highest value derives from analyzes restricted to predated nests, which likely overestimate true frequency (Kaska and Downie 1999). Most other studies report substantially lower values (≤ 0.08%; Fowler 1979; Bárcenas‐Ibarra et al. 2015; Köhnk et al. 2021). Similar low frequencies occur in loggerhead turtles (~0.01%; Drennen 1990; Piovano et al. 2011), olive ridley turtles (0.02%–0.14%; Bárcenas‐Ibarra and Maldonado‐Gasca 2009; Sosa‐Cornejo et al. 2022), and hawksbill turtles (~0.01%; Bárcenas‐Ibarra et al. 2015). Data from Kemp's ridley turtle ( *Lepidochelys kempii* ) further support this pattern (~0.06%; Bárcenas‐Ibarra unpublished data). All frequencies reported here are expressed relative to the total number of eggs analyzed in each study. Since 1998, only one previous occurrence of twin embryos has been documented during sea turtle monitoring at Guanahacabibes (Azanza et al. 2008). The present clutch therefore represents the second documented occurrence of polyembryony at this rookery and the first involving triplet embryos. These data show that while polyembryony recurs across sea turtle taxa, it remains an exceptionally infrequent developmental outcome.

Given this low frequency, it is essential to distinguish true embryonic splitting from egg‐level anomalies. A triple‐yolked egg reported in a wild hawksbill nest showed no embryonic development and did not produce multiple embryos (Lavigne et al. 2025), demonstrating that atypical yolk formation can occur independently of polyembryogenesis. In amniotes, each embryo establishes its own yolk sac vasculature; accordingly, the presence of multiple independent vitelline vascular networks associated with a single yolk mass is consistent with early embryonic splitting (Ewert 1985; Miller 1985). This criterion supports the interpretation of the present embryos from eggs B and C as monozygotic polyembryony rather than multiple fertilization or secondary fusion. Triplet embryos represent an even rarer extension of this process. Most polyembryonic embryos in sea turtles fail to reach hatching, particularly when multiple embryos share a single yolk mass (Fowler 1979; Drennen 1990; Kaska and Downie 1999; Piovano et al. 2011; Bárcenas‐Ibarra and Maldonado‐Gasca 2009; Bárcenas‐Ibarra et al. 2015; Köhnk et al. 2021; Sosa‐Cornejo et al. 2022). The formation of three embryos implies additional or sequential splitting events during early development, increasing developmental complexity and energetic demands within a single egg. Under these conditions, shared access to a finite yolk supply creates an intrinsic resource limitation and promotes asymmetric sibling competition, constraining growth and leading to uneven development (Du and Shine 2022). Such developmental and energetic constraints are expected to increase the likelihood of asymmetry, growth restriction, and congenital malformations in polyembryonic systems (Eckhardt 2018; Vega and Siroski 2021; Cunha et al. 2024; Santiago et al. 2025). This framework may explain the pronounced size disparity and craniofacial anomalies observed in embryo C3. Comparable developmental asymmetry has been reported in polyembryonic cases from other reptilian taxa, including the wild Burmese python, the yellow‐spotted Amazon river turtle, and the lace monitor ( *Varanus varius* ; Eckhardt 2018; Cunha et al. 2024; Santiago et al. 2025).

From an evolutionary perspective, polyembryony in vertebrates is widely regarded as a developmental byproduct rather than an adaptive reproductive strategy (Craig et al. 1997; Avise 2015). The present case is therefore best interpreted as an incidental outcome of early cleavage irregularities. In vertebrates, monozygotic twins originate through early cleavage‐stage separation of the embryo; when separation occurs later, incomplete division or cell lineage interactions may produce asymmetrical or conjoined forms (Saperstein 1981). According to the developmental staging of sea turtles described by Miller (1985), embryos continue development in the nest after stage 6, when the primitive streak is already established and the anteroposterior (craniocaudal) axis has been configured, suggesting that the embryonic splitting observed here occurred earlier in development, prior to oviposition.

Triplet embryos in reptiles remain exceedingly rare and are documented primarily as isolated observations. Notably, most non‐chelonian reptile cases originate under artificial or semi‐controlled conditions. In lizards, including varanids, triplets have been recorded almost exclusively in captive or controlled incubation environments (Krauss and Horn 2004; Mendyk 2007; Eckhardt 2018; Nečas et al. 2020). In snakes, triplets have also been reported under captive conditions, with only a single confirmed wild nest of the Burmese python ( *Python molurus bivittatus* ; Santiago et al. 2025). In crocodilians, triplets involve eggs collected in the wild but incubated artificially (Vega and Siroski 2021). Thus, naturally incubated reptilian triplets remain exceptionally uncommon, underscoring the significance of this observation.

The occurrence of polyembryony in this nest was not accompanied by reduced overall nest viability. Hatching success in the present nest (91.6%) was comparable to values previously reported for green turtle nests in Guanahacabibes, where mean hatching success was 91.07% ± 5.33% at La Barca Beach and 94.02% ± 3.25% at Antonio Beach (Calderón‐Peña and Azanza‐Ricardo 2021). The mean incubation temperature recorded in a nearby nest located 8 m from the study nest (31.87°C ± 1.03°C) was comparable to mean incubation temperatures previously reported at La Barca (31.59°C ± 0.61°C) and Antonio (31.04°C ± 0.49°C) beaches (Calderón‐Peña and Azanza‐Ricardo 2021). Hatchling performance further supported normal nest viability (self‐righting: 2.1 ± 0.8 s; locomotion: 6.9 ± 1.4 m/min), with locomotor values within the range reported by Ischer et al. (2009) (5.16–9.42 m/min). Self‐righting time was shorter and locomotor performance was higher than the values reported by Gatto and Reina (2020) (7.8–10.77 s and 3.33–3.84 m/min, respectively). Despite the high overall nest viability observed, the simultaneous occurrence of a malformed single embryo, polyembryonic twins, and polyembryonic triplets within the same nest is particularly noteworthy given the exceptional rarity of polyembryony in sea turtles.

Sea turtle embryos develop within nests where environmental conditions strongly influence developmental rate, survivorship, sex determination, and the incidence of congenital malformations (Ackerman 1997; Booth and Astill 2001; Bárcenas‐Ibarra et al. 2015; Tomillo and Spotila 2020; Calderón‐Peña and Azanza‐Ricardo 2021). Elevated or fluctuating temperatures can affect embryonic development and survival and have been associated with developmental abnormalities, altered developmental rates, and reduced hatching or emergence success (Miller et al. 2003, 2017; Gammon et al. 2020; Calderón‐Peña and Azanza‐Ricardo 2021; Du and Shine 2022). In Guanahacabibes, Calderón‐Peña and Azanza‐Ricardo (2021) found a significant positive relationship between temperature during the last third of embryonic development and the frequency of hatchlings with supernumerary scutes (*r* = 0.70, *p* < 0.01), whereas no significant relationship was detected between temperature and carapace malformations. Although thermal conditions can influence embryonic development and the expression of congenital abnormalities (Miller et al. 2003; Calderón‐Peña and Azanza‐Ricardo 2021; Du and Shine 2022), the developmental timing described above suggests that incubation temperature would be unlikely to have initiated the embryonic splitting observed here. Temperature‐dependent sex determination is well established in sea turtles, with sex ratios progressively shifting from male‐ to female‐biased as incubation temperatures increase across the transitional range of temperatures (TRT), within which both sexes are produced (Wibbels 2003). In green turtles, the pivotal temperature—defined as the constant incubation temperature at which a 1:1 hatchling sex ratio is expected (Mrosovsky and Pieau 1991)—has been estimated at approximately 29.5°C for nesting populations in Cuba and Suriname (Calderón‐Peña et al. 2020; Godfrey and Mrosovsky 2006). Calderón‐Peña et al. (2020) reported mean nest temperatures exceeding 30°C during most years of a six‐year study in Guanahacabibes, with estimated female hatchling production exceeding 90%. Consistent with previously estimated strongly female‐biased sex ratios for this rookery, all three embryos from Egg C were identified as females based on histological ovarian criteria. Moreover, to our knowledge, this study represents the first report of gonadal sex determination based on histological analysis in naturally occurring polyembryonic embryos preserved initially in ethanol, a rare reproductive context in which embryonic sex determination has seldom been documented.

Taken together, the coexistence of high overall hatching success with multiple congenital malformations and polyembryonic embryos within a single nest demonstrates that rare embryological events can occur without compromising overall nest viability. The present case provides insight into the developmental limits of the amniote embryo and highlights the exceptional nature of multi‐embryo development under natural incubation conditions.

## 5 Conclusions

This study expands the known spectrum of developmental variation in *Chelonia mydas* by documenting a rare case of polyembryonic triplet embryos in a wild sea turtle nest. The integration of detailed morphological assessment, developmental staging, yolk sac vascular characterization, and histological sex identification provides one of the most comprehensive embryological accounts of polyembryony in chelonians under fully natural incubation conditions.

The presence of independent vitelline vascular networks associated with a single yolk mass offers strong embryological evidence of early embryonic splitting, supporting the interpretation of these embryos as cases of monozygotic polyembryony. Histological examination further revealed that all three embryos developed ovarian‐type gonads, providing direct evidence of sex differentiation in polyembryonic sea turtle embryos. Such documentation is rare in reptilian polyembryony and, to our knowledge, represents the first histologically confirmed report of gonadal sex in polyembryonic sea turtle embryos recovered from a natural nest. The observed developmental asymmetry and congenital malformations are consistent with the structural and energetic constraints associated with multi‐embryo development within a single egg.

Beyond its embryological significance, this case highlights the value of long‐term nest‐monitoring programs for detecting rare developmental events that would otherwise remain undocumented. Continued integrative embryological research under natural incubation conditions will be essential for clarifying how such uncommon phenomena occur and for defining the limits of developmental plasticity in wild sea turtle populations.

## Author Contributions

**Julia Azanza‐Ricardo:** conceptualization (equal), data curation (equal), formal analysis (equal), funding acquisition (equal), investigation (equal), methodology (equal), project administration (equal), resources (equal), supervision (equal), writing – original draft (equal), writing – review and editing (equal). **Annelisse Bárcenas‐Ibarra:** conceptualization (equal), data curation (equal), formal analysis (equal), funding acquisition (equal), investigation (equal), methodology (equal), project administration (equal), resources (equal), supervision (equal), writing – original draft (equal). **Kevin Armando Domínguez‐León:** investigation (equal), methodology (equal), writing – review and editing (equal). **Randy Calderón‐Peña:** investigation (equal), methodology (equal), writing – review and editing (equal). **Yunaisy Guerrero‐Esquijarosa:** investigation (equal), methodology (equal), writing – review and editing (equal). **Eduardo E. Colin‐Hidalgo:** investigation (equal), methodology (equal), writing – review and editing (equal). **José A. Marmolejo‐Valencia:** methodology (equal), writing – review and editing (equal). **Horacio Merchant‐Larios:** methodology (equal), writing – review and editing (equal). **Daniel J. Sánchez‐Ochoa:** funding acquisition (equal), methodology (equal), resources (equal), writing – review and editing (equal). **Horacio de la Cueva:** supervision (equal), writing – review and editing (equal).

## Funding

This work was supported by the Guanahacabibes National Park Administration and the University of Havana Sea Turtle Conservation Program. Field research was funded by the Billion Baby Turtles Grant of SEE turtles, the Ocean Foundation, and Sea Turtle Conservancy.

## Conflicts of Interest

The authors declare no conflicts of interest.

## Data Availability

The data that support the findings of this study are available at https://doi.org/10.5281/zenodo.19459549.

## References

[ece374382-bib-0001] Ackerman, R. A. 1997. “The Nest Environment and the Embryonic Development of Sea Turtles.” In The Biology of Sea Turtles, edited by P. L. Lutz and J. A. Musick , vol. 1, 83–106. CRC Press.

[ece374382-bib-0002] Avise, J. C. 2012. “Clones, Hermaphrodites and Pregnancies: Nature's Oddities Offer Evolutionary Lessons on Reproduction.” Journal of Zoology 286, no. 1: 1–14. 10.1111/j.1469-7998.2011.00869.x.

[ece374382-bib-0003] Avise, J. C. 2015. “Evolutionary Perspectives on Clonal Reproduction in Vertebrate Animals.” Proceedings of the National Academy of Sciences of the United States of America 112, no. 29: 8867–8873. 10.1073/pnas.1501720112. PMC4517198 26195735

[ece374382-bib-0004] Azanza, J. , M. E. Ibarra , J. Hernández , R. Díaz , and N. Hernández . 2008. “Análisis de Nidos de Tortuga Verde ( *Chelonia mydas* ) Durante la Temporada 2006 en la Península de Guanahacabibes, Cuba.” Revista de Investigaciones Marinas 29, no. 2: 161–169.

[ece374382-bib-0005] Bárcenas‐Ibarra, A. , H. de la Cueva , I. Rojas‐Lleonart , et al. 2015. “First Approximation to Congenital Malformation Rates in Embryos and Hatchlings of Sea Turtles.” Birth Defects Research Part A: Clinical and Molecular Teratology 103, no. 3: 203–224. 10.1002/bdra.23342. 25761253

[ece374382-bib-0006] Bárcenas‐Ibarra, A. , and A. Maldonado‐Gasca . 2009. “Malformaciones en Embriones y Neonatos de Tortuga Golfina ( *Lepidochelys olivacea* ) en Nuevo Vallarta, Nayarit, México.” Veterinaria México 40: 371–380.

[ece374382-bib-0007] Booth, D. T. , and K. Astill . 2001. “Temperature Variation Within and Between Nests of the Green Sea Turtle, *Chelonia mydas* (Chelonia: Cheloniidae) on Heron Island, Great Barrier Reef.” Australian Journal of Zoology 49: 71–84. 10.1071/ZO00077.

[ece374382-bib-0008] Booth, D. T. , R. Feeney , and Y. Shibata . 2013. “Nest and Maternal Origin Can Influence Morphology and Locomotor Performance of Hatchling Green Turtles ( *Chelonia mydas* ) Incubated in Field Nests.” Marine Biology 160, no. 1: 127–137. 10.1007/s00227-012-2070-y.

[ece374382-bib-0009] Calderón‐Peña, R. , and J. Azanza‐Ricardo . 2021. “Incubation Temperatures, Hatching Success and Congenital Anomalies in Green Turtle Nests From the Guanahacabibes Peninsula, Cuba.” Aquatic Research 4, no. 4: 321–330. 10.3153/AR21027.

[ece374382-bib-0010] Calderón‐Peña, R. , R. Betancourt‐Avila , E. Rodríguez‐Fajardo , Y. Martínez‐González , and J. Azanza‐Ricardo . 2020. “Sex Ratio of the Green Sea Turtle *Chelonia mydas* (Testudines: Cheloniidae) Hatchlings in the Guanahacabibes Peninsula, Cuba.” Revista de Biología Tropical 68, no. 3: 777–784. 10.15517/rbt.v68i3.37955.

[ece374382-bib-0011] Craig, S. F. , L. B. Slobodkin , G. A. Wray , and C. H. Biermann . 1997. “The Paradox of Polyembryony: A Review of Cases and Hypotheses for Its Evolution.” Evolutionary Ecology 11, no. 2: 127–143. 10.1023/A:1018443714917.

[ece374382-bib-0012] Cunha, F. A. G. , M. B. Castro , and F. B. R. Gomes . 2024. “Twin and Triplet Hatchlings in *Podocnemis unifilis* .” Herpetological Review 55, no. 2: 254–256.

[ece374382-bib-0013] Drennen, D. 1990. “Twinning in Loggerhead Sea Turtles ( *Caretta caretta* ).” Marine Turtle Newsletter 48: 19–20.

[ece374382-bib-0014] Du, W.‐G. , and R. Shine . 2022. “The Behavioural and Physiological Ecology of Embryos: Responding to the Challenges of Life Inside an Egg.” Biological Reviews 97, no. 4: 1272–1286. 10.1111/brv.12841. 35166012

[ece374382-bib-0015] Eckert, K. L. 1990. “Twinning in Leatherback Sea Turtle ( *Dermochelys coriacea* ) Embryos.” Journal of Herpetology 24: 317. 10.2307/1564404.

[ece374382-bib-0016] Eckhardt, F. S. 2018. “Triplet From a Single Egg in the Panther Chameleon ( *Furcifer pardalis* ).” Herpetology Notes 11: 777–779.

[ece374382-bib-0017] Ewert, M. A. 1985. “Embryology of Turtles.” In Biology of the Reptilia, edited by C. Gans and F. Billet , vol. 14, 75–267. Wiley.

[ece374382-bib-0018] Feitosa, A. F. , R. H. de Carvalho , R. C. Rufino , O. S. Duarte , and C. V. Feitosa . 2025. “Abnormalities in Hawksbill Sea Turtle Hatchlings, *Eretmochelys imbricata* (Linnaeus, 1766), in Northeastern Brazil.” Herpetology Notes 18: 931–939.

[ece374382-bib-0019] Fowler, L. E. 1979. “Hatching Success and Nest Predation in the Green Sea Turtle, *Chelonia mydas* , at Tortuguero, Costa Rica.” Ecology 60: 946–955. 10.2307/1936863.

[ece374382-bib-0020] Gammon, M. , S. Fossette , G. McGrath , and N. Mitchell . 2020. “A Systematic Review of Metabolic Heat in Sea Turtle Nests and Methods to Model Its Impact on Hatching Success.” Frontiers in Ecology and Evolution 8: 556379. 10.3389/fevo.2020.556379.

[ece374382-bib-0021] Gatto, C. R. , and R. D. Reina . 2020. “Sea Turtle Hatchling Locomotor Performance: Incubation Moisture Effects, Ontogeny and Species‐Specific Patterns.” Journal of Comparative Physiology B 190: 779–793. 10.1007/s00360-020-01307-z. 32959084

[ece374382-bib-0022] Godfrey, M. H. , and N. Mrosovsky . 2006. “Pivotal Temperature for Green Sea Turtles, *Chelonia mydas* , Nesting in Suriname.” Herpetological Journal 16, no. 1: 51–61.

[ece374382-bib-0023] Ischer, T. , K. Ireland , and D. T. Booth . 2009. “Locomotion Performance of Green Turtle ( *Chelonia mydas* ) Hatchlings From Nests at Different Temperatures.” Marine Biology 156: 1399–1409. 10.1007/s00227-009-1180-7.

[ece374382-bib-0024] Iwabuchi, K. 2019. “Overview of Polyembryony.” In Polyembryonic Insects. Entomology Monographs. Springer.

[ece374382-bib-0025] Kaska, Y. , and R. Downie . 1999. “Embryological Development of Sea Turtles ( *Chelonia mydas* , *Caretta caretta* ) in the Mediterranean.” Zoology in the Middle East 19, no. 1: 55–69. 10.1080/09397140.1999.10637796.

[ece374382-bib-0026] Köhnk, S. , R. Brown , and A. Liddell . 2021. “Finding of a Two‐Headed Green Turtle Embryo During Nest Monitoring in Baa Atoll.” Onderstepoort Journal of Veterinary Research 88, no. 1: a1940. 10.4102/ojvr.v88i1.1940. PMC8424721 34476952

[ece374382-bib-0027] Krauss, P. , and H. G. Horn . 2004. “Triplet Lace Monitors ( *Varanus varius* ) Hatching From One Egg.” Reptiles Australia 1, no. 4: 14–15.

[ece374382-bib-0028] Lavigne, A. , R. Bullock , N. J. Shah , C. Tagg , A. Zora , and N. Hemmings . 2025. “Understanding Early Reproductive Failure in Turtles and Tortoises.” Animal Conservation 28, no. 3: 353–364. ISSN 1367–9430. 10.1111/acv.12986.

[ece374382-bib-0029] Marchal, P. 1904. “Recherches sur la biologie et le développement des Hyménoptères parasites. I. La polyembryonie spécifique ou germinogonie.” Archives de Zoologie Expérimentale et Générale 2: 247–335.

[ece374382-bib-0030] Mendyk, R. W. 2007. “Dizygotic Twinning in the Blue Tree Monitor, *Varanus macraei* .” Biawak 1, no. 1: 26–28.

[ece374382-bib-0031] Merchant‐Larios, H. 1999. “Determining Hatchling Sex.” In Research and Management Techniques for the Conservation of Sea Turtles, edited by K. L. Eckert , K. A. Bjorndal , F. A. Abreu‐Grobois , and M. Donnelly . IUCN/SSC Marine Turtle Specialist Group Publication.

[ece374382-bib-0032] Miller, J. , D. Limpus , and M. Godfrey . 2003. “Nest Site Selection, Oviposition, Eggs, Development, Hatching and Emergence of Loggerhead Turtles.” In Loggerhead Sea Turtles, edited by A. B. Bolten and B. E. Witherington , 125–143. Smithsonian Books.

[ece374382-bib-0033] Miller, J. D. 1985. “Embryology of Marine Turtles.” In Biology of the Reptilia, edited by C. Gans and F. Billet , vol. 14, 269–328. Wiley.

[ece374382-bib-0034] Miller, J. D. 1999. “Determining Clutch Size and Hatching Success.” In Research and Management Techniques for the Conservation of Sea Turtles, edited by K. L. Eckert , K. A. Bjorndal , F. A. Abreu‐Grobois , and M. Donnelly , 124–129. IUCN/SSC Marine Turtle Specialist Group.

[ece374382-bib-0035] Miller, J. D. , J. A. Mortimer , and C. J. Limpus . 2017. “A Field Key to the Developmental Stages of Marine Turtles (Cheloniidae) With Notes on the Development of *Dermochelys* .” Chelonian Conservation and Biology 16, no. 2: 111–122. 10.2744/CCB-1261.1.

[ece374382-bib-0036] Monden, C. , G. Pison , and J. Smits . 2021. “Twin Peaks: More Twinning in Humans Than Ever Before.” Human Reproduction 36, no. 6: 1666–1673. 10.1093/humrep/deab029. PMC8129593 33709110

[ece374382-bib-0037] Mrosovsky, N. , and C. Pieau . 1991. “Transitional Range of Temperature, Pivotal Temperatures, and Thermosensitive Stages for Sex Determination in Reptiles.” Amphibia‐Reptilia 12, no. 2: 169–179. 10.1163/156853891X00149.

[ece374382-bib-0038] Nečas, P. , S. Prokopiev , C. Durbin , J. Van Overbeke , and C. Roth . 2020. “Quadruplets, Triplets and Twins in Chameleons (Sauria: Chamaeleonidae).” Archaius 1: 18–23.

[ece374382-bib-0039] Patterson, J. T. 1927. “Polyembryony in Animals.” Quarterly Review of Biology 2, no. 3: 399–426. https://www.jstor.org/stable/2808324.

[ece374382-bib-0040] Phillott, A. D. , and M. H. Godfrey . 2020. “Assessing the Evidence of ‘Infertile’ Sea Turtle Eggs.” Endangered Species Research 41: 329–338. 10.3354/esr01032.

[ece374382-bib-0041] Piovano, S. , Y. Kaska , E. Prazzi , S. Nannarelli , and C. Giacoma . 2011. “Low Incidence of Twinning in the Loggerhead Sea Turtle.” Folia Zoologica 60, no. 2: 159–166. 10.25225/fozo.v60.i2.a10.2011.

[ece374382-bib-0042] Pritchard, P. C. H. , and J. A. Mortimer . 1999. “Taxonomy, External Morphology, and Species Identification.” In Research and Management Techniques for the Conservation of Sea Turtles, edited by K. L. Eckert , K. A. Bjorndal , F. A. Abreu‐Grobois , and M. Donnelly , 21–38. IUCN/SSC Marine Turtle Specialist Group.

[ece374382-bib-0043] Rodríguez‐Cabrera, T. M. , A. H. Gómez , and J. Torres . 2022. “First Recorded Birth of Suspected Non‐Identical Twins in the Cuban boa *Chilabothrus angulifer* .” Herpetological Bulletin 160: 31–34. 10.33256/hb160.3134.

[ece374382-bib-0044] Santiago, G. A. , J. E. Baird‐Lujano , J. C. Guzy , et al. 2025. “First Record of Twin and Triplet Embryos Found in the Clutch of a Wild Burmese python in Southern Florida.” Ecology and Evolution 15, no. 8: e72040. 10.1002/ece3.72040. PMC12378016 40873724

[ece374382-bib-0045] Saperstein, G. 1981. “Diprosopus in a Hereford Calf.” Veterinary Record 108: 234–235. 10.1136/vr.108.11.234 7257121

[ece374382-bib-0046] Shine, R. 1985. “The Evolution of Viviparity in Reptiles: An Ecological Analysis.” In Biology of the Reptilia, edited by C. Gans and F. Billet , vol. 15, 605–694. Wiley.

[ece374382-bib-0047] Sosa‐Cornejo, I. , R. Martín‐Del‐Campo , J. A. González‐Flores , et al. 2022. “Leucism: The Prevalent Congenital Malformation in the Olive Ridley Sea Turtle of Northwestern Mexico.” Diseases of Aquatic Organisms 152: 61–71. 10.3354/dao03705. 36394141

[ece374382-bib-0048] Tomillo, P. S. , and J. R. Spotila . 2020. “Temperature‐Dependent Sex Determination in Sea Turtles in the Context of Climate Change: Uncovering the Adaptive Significance.” BioEssays 42, no. 11: 2000146. 10.1002/bies.202000146. 32896903

[ece374382-bib-0049] Tucker, J. K. , and F. J. Janzen . 1997. “Incidence of Twinning in Turtles.” Copeia 1997: 166–173. 10.2307/1447852.

[ece374382-bib-0050] Vega, G. G. , and P. Siroski . 2021. “Triplets in *Caiman yacare* .” Herpetological Review 52, no. 3: 568–569.

[ece374382-bib-0051] Wibbels, T. 2003. “Critical Approaches to Sex Determination in Sea Turtles.” In The Biology of Sea Turtles, edited by P. L. Lutz , J. A. Musick , and J. Wyneken , vol. 2, 103–134. CRC Press.

